# Targeting of a Conserved Epitope in Mouse and Human GPVI Differently Affects Receptor Function

**DOI:** 10.3390/ijms23158610

**Published:** 2022-08-03

**Authors:** Stefano Navarro, Andreas Starke, Johan W. M. Heemskerk, Marijke J. E. Kuijpers, David Stegner, Bernhard Nieswandt

**Affiliations:** 1 Institute of Experimental Biomedicine, University Hospital Würzburg and Rudolf Virchow Center for Integrative and Translational Bioimaging, Josef-Schneider-Straße 2, 97080 Würzburg, Germany; navarro_s@ukw.de (S.N.); anstarke@web.de (A.S.); stegner@virchow.uni-wuerzburg.de (D.S.); 2 Department of Biochemistry, Cardiovascular Research Institute Maastricht (CARIM), Maastricht University, 6229 ER Maastricht, The Netherlands; jwmheem722@outlook.com (J.W.M.H.); marijke.kuijpers@maastrichtuniversity.nl (M.J.E.K.); 3 Synapse Research Institute, Kon. Emmaplein 7, 6214 AC Maastricht, The Netherlands; 4 Thrombosis Expertise Center, Heart and Vascular Center, Maastricht University Medical Center+, Professor Debyelaan 25, 6229 HX Maastricht, The Netherlands

**Keywords:** glycoprotein VI, JAQ1, platelet receptors, platelet activation, platelet inhibition

## Abstract

Glycoprotein (GP) VI is the major platelet collagen receptor and a promising anti-thrombotic target. This was first demonstrated in mice using the rat monoclonal antibody JAQ1, which completely blocks the Collagen-Related Peptide (CRP)-binding site on mouse GPVI and efficiently inhibits mouse platelet adhesion, activation and aggregation on collagen. Here, we show for the first time that JAQ1 cross-reacts with human GPVI (huGPVI), but not with GPVI in other tested species, including rat, rabbit, guinea pig, swine, and dog. We further demonstrate that JAQ1 differently modulates mouse and human GPVI function. Similar to its effects on mouse GPVI (mGPVI), JAQ1 inhibits CRP-induced activation in human platelets, whereas, in stark contrast to mouse GPVI, it does not inhibit the adhesion, activation or aggregate formation of human platelets on collagen, but causes instead an increased response. This effect was also seen with platelets from newly generated human GPVI knockin mice (*hGP6^tg/tg^*). These results indicate that the binding of JAQ1 to a structurally conserved epitope in GPVI differently affects its function in human and mouse platelets.

## 1. Introduction

Platelets are small, anucleated blood cells produced by bone marrow-resident megakaryocytes (MK), that have key roles in hemostasis and thrombosis [1,2]. At the sites of vascular injury, the platelets recognize and bind to specific ligands on the exposed extracellular matrix, become activated and aggregate to form a hemostatic plug that seals the vessel and prevents excessive blood loss. However, in pathological conditions, the intravascular platelet activation can precipitate the formation of vessel-occluding thrombi, leading to ischemic disease states, such as stroke or myocardial infarction [3,4]. Therefore, anti-platelet drugs have become indispensable therapeutics to efficiently prevent or treat arterial thrombosis, but they carry an inherent risk of bleeding, most obviously in multimorbid patients requiring dual platelet inhibition or combined anticoagulation [5]. Among the major platelet receptors, glycoprotein (GP) VI has emerged as a promising therapeutic target, as its functional inhibition or genetic deletion provides protection from arterial thrombus formation in vivo, while only minimally affecting hemostasis [6]. GPVI is the main signaling receptor for collagen and is expressed exclusively on platelets and MK. GPVI is associated with the FcR γ (Fc receptor γ)-chain, which is responsible for the signaling via its immunoreceptor-tyrosine-based-activation-motif (ITAM). Besides collagen, several additional physiological agonists have been identified in recent years. These include fibrinogen, fibrillar fibrin [7,8,9,10] and fibronectin [11], the basement membrane protein nidogen-1 [12] and laminins [13]. These ligands are likely to—at least in part—contribute to the role of GPVI in pathophysiological processes beyond thrombosis, such as ischemia-reperfusion injury [14], sepsis [15], cancer and metastasis [16,17] and venous thrombosis [18]. Collectively, these studies highlight the potential benefits of efficient anti-GPVI agents. In fact, the first inhibitors of GPVI are now entering the clinic. The GPVI-blocking Fab (ACT017, glenzocimab) is assessed in the context of acute ischemic stroke [19,20,21]. In transgenic mice carrying the human *GP6* gene, glenzocimab was found to be effective in thrombus suppression, without impacting GPVI-dependent inflammatory hemostasis [22]. The first studies on the (patho-)physiological function and in vivo relevance of GPVI were performed in mice and capitalized on the first reported anti-GPVI monoclonal antibody (mAb), JAQ1 (rat IgG2a) [23,24]. It was shown that JAQ1 completely blocks the collagen-related-peptide (CRP)-induced activation of mouse platelets and virtually abolishes mouse platelet adhesion, activation and aggregate formation on collagen [6,25]. Notably, a possible interaction between JAQ1 and human GPVI (huGPVI) has not been studied. Mouse and human GPVI share ~67.3% of their nucleotide sequence and ~64.4% of the amino-acid sequence, with huGPVI having an intracellular domain that is 24 amino acids longer than that of mouse GPVI (mGPVI) [26]. In addition, the extracellular domains of the receptor also differ between the two species, best documented by the ability of huGPVI, but not mGPVI, to support platelet spreading on fibrinogen [27]. As this raises the question as to whether a specific anti-mGPVI antibody could bind huGPVI and thereby modulate its function in a comparable manner [28], we assessed the effects of JAQ1 on huGPVI. Here, we show that JAQ1 recognizes a conformational epitope on huGPVI and efficiently activates the human platelets upon cross-linking by a secondary antibody. Similar to its effect on mGPVI, JAQ1 inhibited the human platelet activation by CRP, but, in stark contrast to mouse platelets, it did not inhibit, but rather promoted the adhesion, activation and aggregate formation of human platelets on collagen. These differential effects of JAQ1 on huGPVI were confirmed in platelets from a newly generated mouse line expressing huGPVI instead of mGPVI.

## 2. Results

### 2.1. Anti-Mouse GPVI Monoclonal Antibody JAQ1 Binds Human GPVI and Modulates Receptor Function

**Figure 1:**
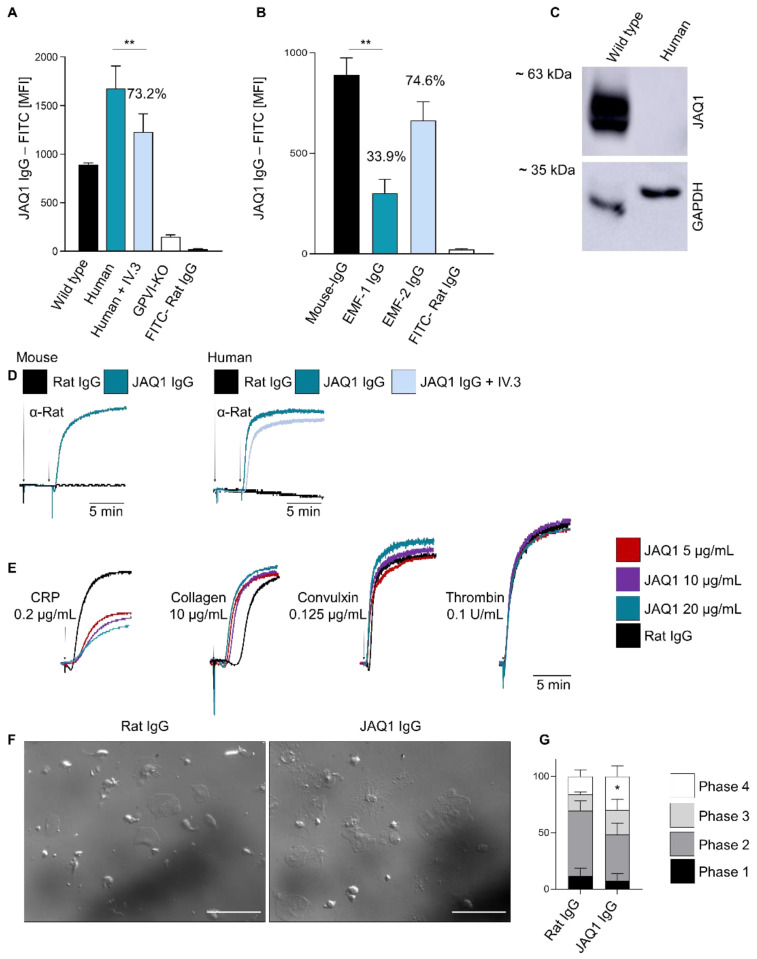
Anti-mouse GPVI monoclonal antibody JAQ1 binds to human GPVI and modulates its function. (**A**) Washed human or mouse blood was pre-incubated with JAQ1 IgG-FITC and Mean Fluorescence Intensity (MFI) was measured by flow cytometry; where indicated, human blood was pre-incubated with 20 µg/mL IV.3. Irrelevant rat-IgG-FITC was used as control; (**B**) Human blood was pre-incubated with 20 µg/mL EMF1, EMF-2 or control IgG for 10 min and subsequently incubated with JAQ1-FITC for 10 min, irrelevant IgG-FITC was used as control; (**C**) Mouse (*WT*) and human platelet lysates were separated by 10% SDS-PAGE under non-reducing condition and blotted onto PVDF membrane. JAQ1-HRP was used to detect GPVI on the membrane. GAPDH served as loading control; (**D**) Murine or human washed platelets were pre-incubated with either JAQ1 or a control IgG and aggregometry was performed; crosslinking of bound antibody was induced by adding an anti-Rat IgG antibody and light transmission was recorded for 15 min. When indicated, human platelets were incubated with IV.3 prior to JAQ1 addition; (**E**) Human washed platelets were pre-incubated with either JAQ1 or a control IgG and aggregometry was performed; aggregation was induced using the indicated agonists and for 10 min; (**F**,**G**) Human-washed platelets were pre-incubated with IV.3 plus JAQ1 IgG or a control IgG and let spread on a 100 µg/mL fibrinogen-coated surface for 45 min at 37 °C. Experiments shown are representative of n = 4. Flow cytometry and spreading data are expressed as mean ± SD, significance is expressed as * *p* < 0.05, ** *p* < 0.01, vs. indicated group (*t*-test).

JAQ1 (rat IgG2a) is the first anti-GPVI mAb reported in the literature and was initially raised against mouse GPVI [24]. JAQ1 completely blocks the major collagen binding site/CRP-binding site in mGPVI [6], resulting in profound inhibition of platelet adhesion, activation and aggregate formation on collagen in vitro [6,24,29]. To assess potential cross-reactivity of JAQ1 with GPVI in other species, we assessed the binding of JAQ1-FITC to platelets in freshly prepared diluted heparinized blood by flow cytometry. In agreement with previous descriptions, JAQ1-FITC robustly bound to the wild type (*WT)*, but not *Gp6^−/−^* mouse platelets (Figure 1A). In addition, we observed no binding to the platelets from closely related species, such as rat, rabbit, guinea pig, swine or dog (Table 1). Remarkably, however, JAQ1-FITC robustly bound to human platelets (Figure 1A, Table 1). Next, we assessed whether JAQ1 binding would be affected by the pre-incubation of human platelets with different, established anti-huGPVI monoclonal antibodies (mAbs). Indeed, JAQ1-FITC binding was reduced by ~66% after pre-incubation with the function-blocking anti-huGPVI mAb EMF-1 [30], but only partially by the non-function-blocking EMF-2 (~26%) (Figure 1B). Subsequently, we tested whether JAQ1 recognizes huGPVI in a Western blot analysis of human platelet lysate. However, while JAQ1 efficiently detected mGPVI no band in the human platelet lysates was observed, indicating that the epitope on huGPVI is conformation sensitive (Figure 1C).

Next, we assessed the effects of JAQ1 on the human platelet aggregation. The cross-linking of JAQ1 with a secondary anti-rat IgG antibody triggered the rapid aggregation of human platelets, similar to mouse platelets (Figure 1D). Of note, this platelet response was not dependent on FcγRIIa, as blocking this with IV.3 antibody did not prevent JAQ1-cross-linking-induced platelet aggregation, but only minimally delayed it (Figure 1D; Figure S1A, Supplementary Materials). The pre-incubation of human-washed platelets with 5, 10 or 20 µg/mL JAQ1 reduced and delayed aggregation in response to CRP, but in contrast to mouse platelets (as shown in paragraph 2.3) this was not abrogated. Notably, the traces of the JAQ1-treated samples showed a reduced platelet-shape change, pointing to a JAQ1-dependent platelet pre-activation and indicating that the residual observable aggregation is partly due to this effect (Figure 1E; Figure S1B,C, Supplementary Materials). Intriguingly, the presence of JAQ1 rather promoted collagen- and convulxin- dependent aggregate formation, and this occurred independently of FcγRIIa (Figure 1E; Figure S1B,C, Supplementary Materials), indicating that JAQ1 modulates huGPVI towards a pre-active state. The treatment with higher concentrations of JAQ1 slightly exacerbated the CRP-inhibitory effect and convulxin-dependent increased aggregation, but not with collagen (Figure 1E; Figure S1B,C, Supplementary Materials). Finally, we tested the JAQ1 effect on the spreading of human platelets on a fibrinogen-coated surface in the absence of additional agonists. Interestingly, pre-incubation with JAQ1 but not the control IgG increased the percentage of the fully spread platelets (phase 4) on the surface (Figure 1F,G). Collectively, these data indicate that the binding epitope of JAQ1 may functionally not be fully conserved between mouse and human GPVI.

### 2.2. A humanized GP6 Mouse Line to Study the Effect of JAQ1 on huGPVI

**Figure 2:**
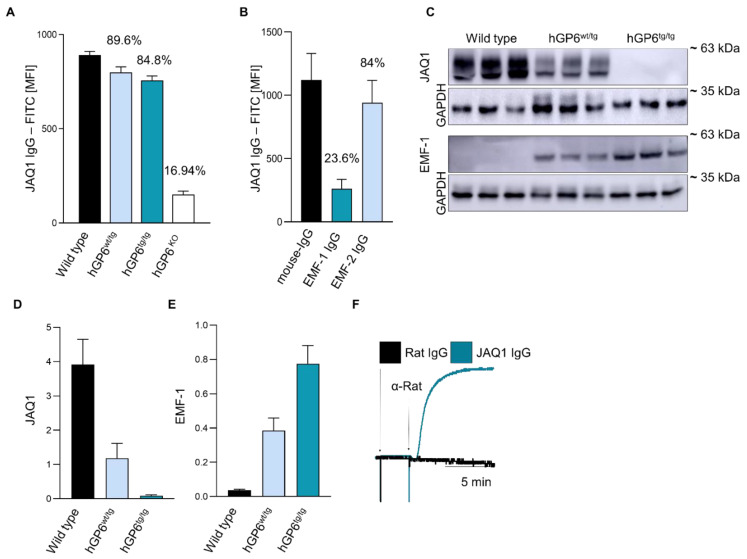
*hGP6^tg/tg^* mice confirm that JAQ1 binds to native human GPVI on the platelet surface, but not in Western blot analysis. (**A**) *WT*, *hGP6^wt/tg^*, *hGP6^tg/tg^* and *Gp6^−/−^*-washed blood was incubated JAQ1-FITC and MFI was measured by flow cytometry; (**B**) *hGP6^tg/tg^*-washed blood was pre-incubated with either EMF-1, EMF-2 or control IgG and subsequently incubated with JAQ1-FITC; (**C**) *WT*, *hGP6^wt/tg^* and *hGP6^tg/tg^* platelet lysates were separated by SDS-PAGE under non-reducing conditions and blotted onto a PVDF membrane. JAQ1 or EMF-1 were used to detect GPVI on the membrane. GAPDH served as loading control; (**D**,**E**) Western blot quantitative analysis relative to loading control; (**F**) *hGP6^tg/tg^* washed platelets were pre-incubated with JAQ1 or control IgG and aggregate formation was induced using anti-rat IgG antibodies (20 µg/mL). Experiments shown are representative of *n* = 4, Western blot of *n* = 3. Flow cytometry and Western blot data are expressed as means ± SD.

In order to study the JAQ1 effects on huGPVI in the absence of possible FcγRIIa interferences, we capitalized on a newly generated mouse line humanized for the *GP6* gene (*hGP6^tg/tg^*). The mouse line generation is thoroughly described in the Materials and Methods Section 4.3; (Figure S4, Supplementary Materials). To ensure the suitability of the newly generated mouse line for further experiments, we analyzed the platelet count, size and expression levels of prominent membrane glycoproteins and found no alteration compared to the wild-type controls, except for the selective expression of human or mouse GPVI (Tables S1 and S2, Supplementary Materials). As expected, we also did not find any difference in the overall platelet activation (Figure S4A,B, Supplementary Materials) and GPVI-dependent platelet aggregation and thrombus formation under flow (Figure S4C–F, Supplementary Materials). Next, we confirmed by flow cytometry that JAQ1 binds to the platelets of *hGP6^tg/tg^*, *hGP6^wt/tg^* and wild-type mice in a comparable manner (Figure 2A). Furthermore, we tested whether EMF-1 or EMF-2 compete with JAQ1 for binding to the *hGP6^tg/tg^* platelets. In line with the results obtained with the human platelets, the JAQ1-FITC binding was profoundly reduced upon pre-incubating the platelets with EMF-1 (~76.4% reduction), but very marginally by EMF-2 (~16%) (Figure 2B). To exclude that the inability of JAQ1 to recognize human GPVI in a Western blot analysis was due to species-specific glycosylation, we probed the platelet lysates from the *WT*, *hGP6^tg/tg^* and *hGP6^wt/tg^* animals with JAQ1. As expected, the signals were only obtained in the samples from the *WT* or h*GP6*^wt/tg^ mice, but not from the h*GP6*^tg/tg^ platelet lysates. Of note, less mGPVI was detected in the h*GP6*^wt/tg^ platelet lysates as compared to the *WT* platelets (70% reduction). Likewise, EMF-1 recognized huGPVI in the lysates of *hGP6^tg/tg^* as well as the *hGP6^wt/tg^* platelets (Figure 2C). As expected, the signal obtained from the *hGP6^wt/tg^* lysates was reduced (−49.3%) as compared to that of the *hGP6^tg/tg^* platelet lysates (Figure 2D,E). Finally, pre-incubation with JAQ1 IgG did not induce an evident platelet aggregation, whereas the cross-linking of JAQ1 with a secondary anti-rat IgG antibody induced a rapid aggregate formation of *hGP6^tg/tg^*-derived platelets (Figure 2F; Figure S2, Supplementary Materials). Overall, these data confirm the specific JAQ1 binding to huGPVI and illustrate that the newly generated mouse line is a suitable model for testing the huGPVI-targeting molecules.

### 2.3. Differential Effect of JAQ1 on huGPVI and mGPVI

**Figure 3:**
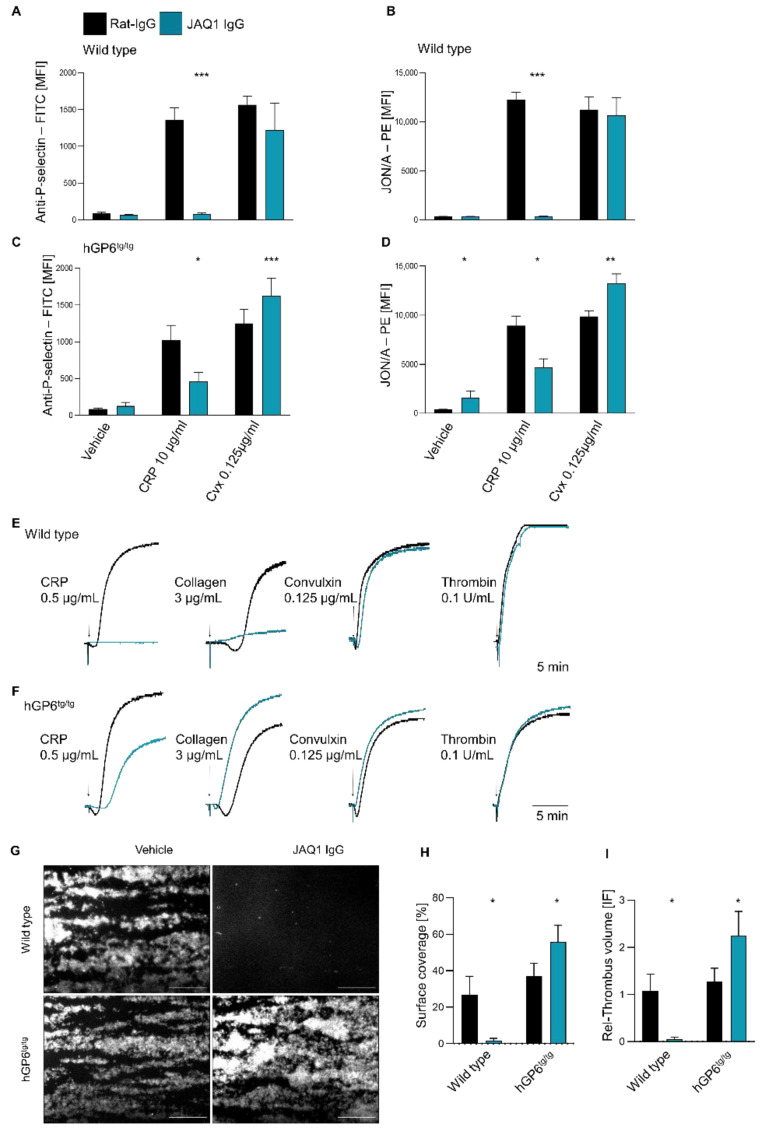
Differential effect of JAQ1 on huGPVI and mGPVI. (**A**–**D**) WT (**A**,**B**) or h*GP6^tg/tg^* (**C**,**D**) diluted heparinized blood was pre-incubated with 20 µg/mL JAQ1 or control-IgG. Treated platelets were incubated with WUG 1.9-FITC (**A**,**C**), JON/A-PE (**B**,**D**) and stimulated with CRP (10 µg/mL), convulxin (1.25 µg/mL) or vehicle; (**E**,**F**) Washed platelets from WT (**E**) or h*GP6^tg/tg^* (**F**) were pre-incubated with 20 µg/mL JAQ1 or control IgG and aggregation was induced with the indicated agonists; aggregation was measured for 10 min; (**G**–**I**) Heparinized WT or h*GP6^tg/tg^* blood was pre-incubated with 20 µg/mL JAQ1 or control IgG and tested in flow adhesion assay on a collagen-coated surface. Percentage of the covered surface (**H**) and relative volume of thrombi (**I**) were analyzed based on fluorescence intensity of anti-GPIX-AF488 derivative. Experiments shown are representative of *n* = 4. Flow cytometry data are expressed as means ± SD, significance is expressed as * *p* < 0.05, ** *p* < 0.01, *** *p* < 0.001 vs. indicated group (*t*-test).

The effect of JAQ1 on the GPVI-mediated platelet activation was assessed by flow cytometry, using *WT* and *hGP6^tg/tg^* platelets. In line with previous reports [6], JAQ1 abrogated the CRP-induced platelet activation of the *WT* platelets, while convulxin (CVX)-induced activation remained intact (Figure 3A,B). In contrast, the *hGP6^tg/tg^* platelets displayed only moderately inhibited CRP-dependent platelet activation, while convulxin-induced integrin activation was even enhanced (Figure 3C,D). Next, the effect of JAQ1 on the platelet aggregation was assessed. On the *WT* platelets, JAQ1 abrogated the CRP-induced and dramatically reduced the collagen-induced aggregation, while aggregation to the convulxin or non-GPVI agonist was unaltered (Figure 3E; Figure S3A,C, Supplementary Materials; and data not shown). Interestingly, the aggregation of the *hGP6^tg/tg^* platelets in response to CRP was delayed and reduced following the JAQ1-pretreatment. In stark contrast, JAQ1 accelerated and fostered the collagen- and convulxin-induced *hGP6^tg/tg^* platelet aggregation (Figure 3F; Figure S3B,D, Supplementary Materials), confirming our results with the human platelets (Figure 1). To assess whether the enhancement of platelet activation and aggregation would result in an accelerated thrombus formation, we tested the effect of JAQ1 on the *WT* and *hGP6^tg/tg^* platelets in a whole mouse blood-flow adhesion assay on a collagen-coated surface. As expected, JAQ1 completely abolished the thrombus formation in the *WT* blood. Strikingly, however, the formation of the thrombi was potently enhanced after incubation of the *hGP6^tg/tg^*-derived blood, thus illustrating the differential effects of JAQ1 on mouse versus human GPVI (Figure 3G–I).

## 3. Discussion

The monoclonal antibody JAQ1 has become a widely used tool for platelet research and its inhibitory effect on mouse GPVI has been thoroughly characterized. Here, we report, for the first time, that this antibody also cross-reacts with huGPVI. Interestingly, however, our data suggest that the binding epitope is functionally different between mGPVI and huGPVI. While JAQ1 inhibits the CRP-dependent platelet activation in both species (albeit at different levels), it differs in its effects on convulxin- and collagen-induced platelet activation (Figure 1, Figure 2 and Figure 3). On the murine platelets, JAQ1 blocks activation in response to low and intermediate collagen concentrations (Figure 3, [38]), while it enhances collagen-induced activation of huGPVI expressing platelets (Figure 1 and Figure 3; Figures S1 and S3, Supplementary Materials). One explanation could be that JAQ1 stabilizes the GPVI dimers (or clusters), which ‘prime’ GPVI for subsequent platelet activation, thereby accelerating and enhancing the platelet activation. Of note, JAQ1 even triggers a pre-activation of the human but not the mouse GPVI, resulting in subtle integrin activation (already in the absence of further agonists) as revealed by flow cytometry (Figure 3A–D).

Very unexpectedly, our data show that JAQ1 binds to a conserved epitope in mouse and human GPVI that somehow evolved to differ in its functional significance. Mouse, rat and guinea pig diverged from human ~90 million years ago [39], and are more closely related within each other with respect to other species, such as dog and swine [40]. These data are in agreement with our flow cytometry results, showing no binding to GPVI on the platelet surface in these animals. The previous studies revealed discrepancies between the mouse and human GPVI affinity to specific ligands and discussed possible differences with regard to the relevance of this receptor in thrombosis [41], thus, underscoring the need to utilize humanized animal models. Indeed, our newly generated mouse line allowed us to faithfully reproduce the results obtained with human platelets, thereby excluding the possible overlapping effects of FcγRIIa, which is present in human but not mouse platelets and signals through a similar pathway as GPVI. These data clearly show that the *hGP6^tg/tg^* mice reported here are a suitable model system for testing GPVI modulators in vitro and in vivo.

In conclusion, we present a previously undescribed and unexpected pre-activating effect of JAQ1 on huGPVI. This study also illustrated how the same epitope in human and mouse GPVI has genetically diverged during evolution, leading to a different functional significance.

## 4. Materials and Methods

### 4.1. Antibodies and Reagents

The collagen-related peptide (CRP) was a generous gift from Paul Bray (Baylor College, USA); Horm collagen was purchased from Takeda (Linz, Austria); convulxin was purchased from Enzo Life Sciences (New York, NY, USA); thrombin was purchased from Roche Diagnostic (Mannheim, Germany); rabbit anti-GAPDH and rat anti-mouse IgG-HRP antibodies were purchased from Sigma-Aldrich (Steinheim, Germany); goat anti-rat IgG-HRP was purchased from Dianova (Hamburg, Germany); anti-rabbit IgG-HRP was purchased from Jackson Immuno (Suffolk, UK). For the human blood collection, the S-monovettes 3.2% citrate and Safety-Fly-Needle 21G were purchased from Sarstedt (Nümbrecht, Germany). The micro-cuvettes for aggregometry were purchased from LABITec (Ahrensburg, Germany). The 5 mL Polystyrene Round-Bottom Tubes for flow cytometry were purchased from Corning Inc. (New York, NY, USA). The heparin was purchased from Ratiopharm (Ulm, Germany). The JAQ1 [24], EMF-1 [30], EMF-2, JON/A [42,43] and WUG 1.9 were generated in house. The IV.3 antibody was purchased from Biozol (Eching, Germany).

### 4.2. Blood Donors and Blood Collection

The blood was obtained from healthy volunteers not undergoing anticoagulant or antiplatelet drug therapy for at least 4 weeks before recruitment. For the present study, the volunteers signed a written informed consent after approval by the Institutional Review Boards of the University of Würzburg and in accordance with the Declaration of Helsinki. The relevant guidelines and regulations were followed during the performance of all of the described methods. Butterfly needles were used for the collection of blood by venipuncture; the samples were collected into 9 mL tubes containing 3.2% trisodium citrate. For all of the studies, the blood was used within 4 h from withdrawal and kept at room temperature.

### 4.3. Animals

The animals used in this study were matched based on genetic background, sex and age. The experiments with animals shown in this article had been previously approved by the district government of Lower Franconia (Regierung von Unterfranken) and performed following the current Animal Research: Reporting of In Vivo Experiments guidelines (https://www.nc3rs.org.uk/arrive-guidelines, accessed on 15 June 2022).In order to generate the humanized *GP6* (*hGP6^tg/tg^*) mouse line, the cDNA-expressing human GPVI (huGPVI) was inserted at the level of the murine ATG of the mouse *Gp6* gene via CRISPR-Cas9 technology. The mutagenesis was carried out on the base of previous publications [44] via inserting the cDNA-expressing huGPVI within the exon 1 of the *WT* gene, thus allowing for the selective expression of the human but not mouse GPVI (Figure S5, Supplementary Materials). The mice were genotyped by PCR with the forward primer 5′-TGGCAAGAAGAGATAAGTGGTGGCT-3′, the reverse primers 5′-CAGGTCACCTTCAGGACTCACCAAT-3′ for the wild-type amplification and 5′-CAGACTTCTCTTCATGGCCGGGAT-3′ for the transgenic mouse amplification. For the experiments, venous blood was collected in 300 µL of 20 U/mL heparin via retro-orbital bleeding.

### 4.4. Measurement of Platelet Count and Size

For the assessment of platelet size and count, the mice were bled into tubes coated with EDTA; the parameters were measured using an automated cell counter (ScilVet, scil animal care company GmbH, Viernheim, Germany).

### 4.5. Washed Human and Murine Platelets

The human-washed platelets were obtained as follows: 2 mL of ACD pH4.5 was added to the citrated blood and centrifuged for 20′ at 300× *g* at room temperature. The Platelet-Rich-Plasma was collected in new 15 mL tubes and supplemented with 1/10 ACD, 2 μL of apyrase/mL (0.02 U mL^−1^; A6410, Sigma-Aldrich) and 5 μL PGI_2_/_mL_ (0.1 μg mL^−1^; P6188, Sigma-Aldrich). The platelets were pelleted by centrifugation for 10 min at 500× *g*, washed twice with Tyrode’s buffer containing 2 μL apyrase/mL and 5 μL PGI_2_/mL. The murine platelets were washed as previously described [45].

### 4.6. Flow Cytometry Assays

The human blood diluted 1:10 and the murine blood diluted 1:20 in Tyrode’s buffer—Ca^2+^ was used for the flow cytometry analysis. For the platelet activation analysis, the murine blood was diluted in Tyrode’s buffer + Ca^2+^. JON/A-PE (Emfret Analytics, Eibelstadt, Germany) was used to detect the activated integrin αIIbβ3 [42] whilst P-selectin exposure was detected with a specific anti-mPselectin FITC-conjugated antibody WUG 1.9 [42]. The diluted murine blood was incubated with either CRP (10 µg/mL), convulxin (1.25 µg/mL), thrombin (0.1 U/mL) or vehicle control, together with JON/A-PE and anti-Psel-FITC for 12 min and finally diluted in 500 µL PBS. The measurement of the MFI was performed on a FACSCelesta (BD Biosciences, Gurugram, India).

### 4.7. Western Blot

The human and murine platelets were lysed at a concentration of 5 × 10^8^/mL in an IP-buffer containing a protease inhibitor cocktail. The platelet lysates were mixed with a loading buffer containing SDS and pre-heated to 95 °C for 5 min before loading in a 10% polyacrylamide gel, and the immunoblotting was performed as previously described [46,47].

### 4.8. Aggregometry Assay

Human- and murine-washed platelets were resuspended at a concentration of 2 × 10^8^/mL in Tyrode’s buffer—Ca^2+^. The aggregometry was performed as previously described [48]. The human or murine platelets were activated using 0.5 µg/mL Collagen-Related-Peptide (CRP), convulxin (0.125 µg/mL) or collagen 3 µg/mL (mouse) and 10 µg/mL (human). The measurements were performed using an APACT 4 aggregometer from LABITec (Ahrensburg, Germany).

### 4.9. Spreading Assay

The human platelets were washed and resuspended at a concentration of 10^8^/mL in Tyrode’s buffer—Ca^2+^. The washed platelets were incubated with either JAQ1 IgG or control rat IgG for 1 min and then pipetted onto a 100 µg/mL fibrinogen-coated surface. The platelets were allowed to spread for 45 min. Fixation, coating and visualization were performed, as previously described [49].

## Figures and Tables

**Table 1:** Flow cytometric analysis of JAQ1 binding to the platelet surface of different species. The data are reported as MFI of JAQ1-FITC (JAQ1 MFI) and as percentages with respect to mouse MFI (JAQ1%). Mean Platelet Volume (MPV) of the different species are reported in femtoliter (MPV). “Negative” indicates MFI values comparable to isotype IgG-FITC control values. Data are reported as mean ± SD.

Species	JAQ1 MFI	MPV (fl)	JAQ1%	MPV Reference
Mouse	995 ± 24.5	5.5–6	100%	[31]
Rat	Negative	4.4–6.9	-	[32]
Rabbit	Negative	5.55–6.35	-	[33]
Guinea Pig	Negative	7.1–8.2	-	[34]
Swine	Negative	8.4–9.75	-	[35]
Dog	Negative	8–12	-	[36]
Human	1296 ± 47.7	9.4–12–3	130%	[37]

## Data Availability

All data are included in the manuscript as figures, tables or Supplementary Materials.

## Supplementary Materials

The supporting information can be downloaded at: https://www.mdpi.com/article/10.3390/ijms23158610/s1.
