# Supplementary material for: Targeting of a Conserved Epitope in Mouse and Human GPVI Differently Affects Receptor Function
Source: Int J Mol Sci. 2022 Aug 3;23(15):8610. doi: 10.3390/ijms23158610 (PMC9369317; doi:10.3390/ijms23158610)
Supplement: Supplementary file 1 [file ijms-23-08610-s001.zip › ijms-1830286-supplementary.pdf]

Online Supplement

Targeting of a conserved epitope in mouse and human GPVI differently affects receptor function

Stefano Navarro [1,2], Andreas Starke[1], Johan W.M. Heemskerk [2,3], Marijke J. E. Kuijpers [2,4], David Stegner [1], Bernhard Nieswandt [1,*]

1 Institute of Experimental Biomedicine, University Hospital Würzburg and Rudolf Virchow Center for Integrative and Translational Bioimaging, Josef-Schneider-Straße 2, 97080 Würzburg, Germany.
2 Department of Biochemistry, Cardiovascular Research Institute Maastricht (CARIM), Maastricht Uni-versity, 6229 ER Maastricht, The Netherlands
3 Synapse Research Institute, Kon. Emmaplein 7, 6214 AC Maastricht, The Netherlands.
4 Thrombosis Expertise Center, Heart and Vascular Center, Maastricht University Medical Center+, Maastricht, Professor Debyelaan 25, 6229HX Maastricht, The Netherlands. Tel. +31-43-3881674.
*Correspondence: bernhard.nieswandt@virchow.uni-wuerzburg.de

Parameter	Wild-type	h*GP6* $^{tg/tg}$	TTEST
Platelet count $10^3/mm^3$	1084 ± 78	1212.75 ± 86.01	ns
Platelet Size (fl)	5.75 ± 0.21	5.8 ± 0.16	ns

Suppl. Table S1. Platelet count and size. EDTA-treated blood was measured with an automated cell counter (ScilVet). Experiments shown are representative of n=5. Flow cytometry data are expressed as means ± SD.

Glycoprotein	Wild-type (MFI)	h*GP6* $^{tg/tg}$ (MFI)	TTEST
GPIb	6337 ± 94.9	6209 ± 207.1	ns
GPIbβ	13072.5 ±523.7	13421 ± 909.1	ns
αIIbβ3	15043.5 ± 475.3	15740 ± 382.8	ns
α2β1	1570.25 ± 67.3	1530.25 ± 22.3	ns
α5β1	1038 ± 69.6	1031 ± 44.9	ns
GPIX	8257 ± 228.1	8336.75 ± 164	ns
β3	8590 ± 639.2	8065.75 ± 212.5	ns
GPV	8140.25 ± 184.5	8366 ± 117.3	ns
CD9	38258.5 ± 335.65	37954.5 ± 476.6	ns
mGPVI	790.5 ± 19.18	86.43 ± 19.19	***
huGPVI	223.5 ± 15.55	838.75 ± 32.81	***

Suppl. Table S2. Flow cytometric analysis of platelet glycoprotein expression. The expression levels of platelet glycoproteins were determined by flow cytometry. Experiments shown are representative of n=5. Data are expressed as means ± SD, significance is expressed as * $p < 0.05$, ** $p < 0.01$, *** $p < 0.001$ vs. indicated group (t-test).

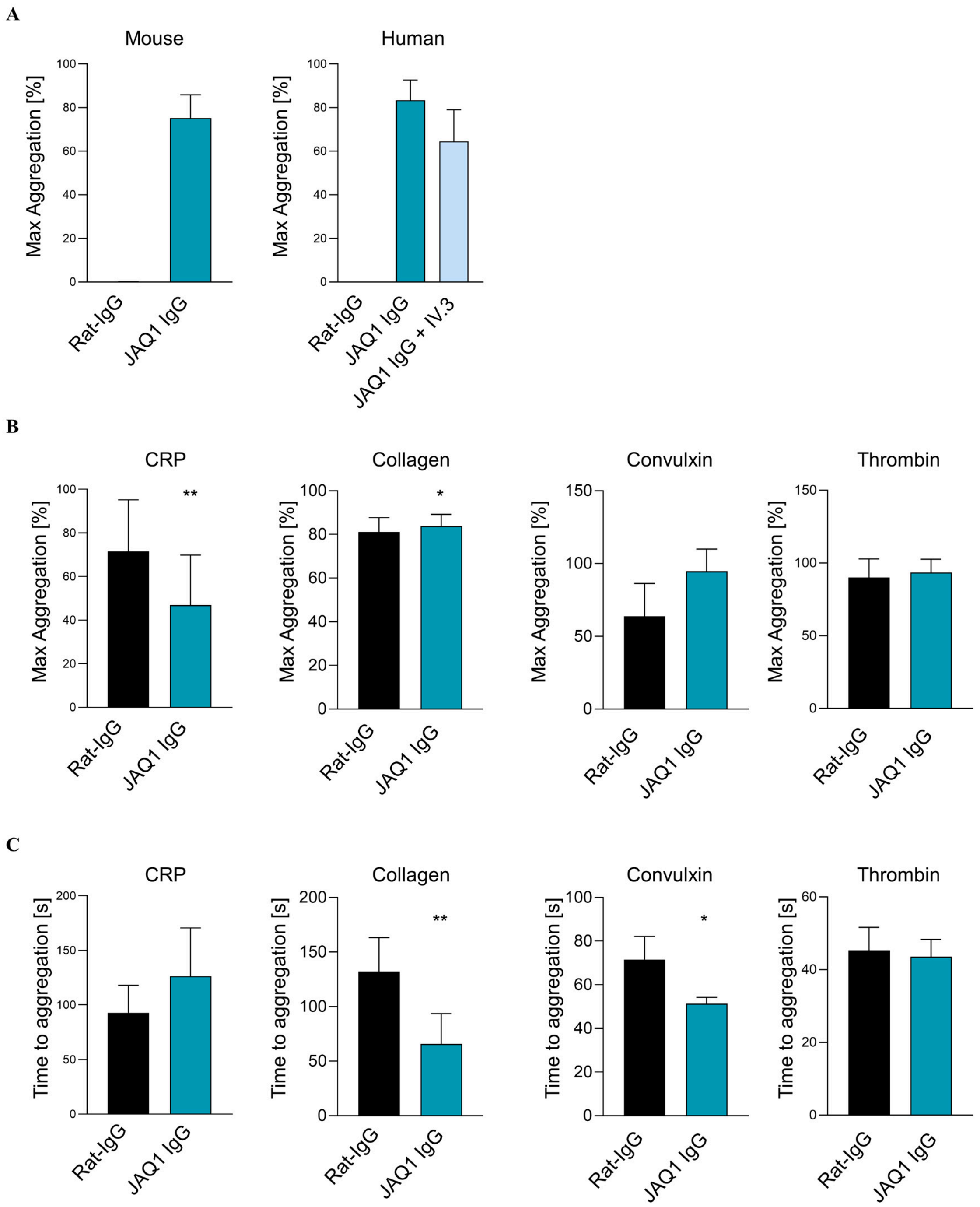

Suppl. Figure S1 . Anti-mouse GPVI monoclonal antibody JAQ1 binds human GPVI and modulates receptor function. Analysis of light transmission aggregometry reported as maximal aggregation and time to aggregation (time to begin of aggregate formation). (A) Maximal aggregation relative to Figure 1D. (B-C) Analysis of aggregation curves shown in Figure 1E and reported as maximal aggregation (B) or time to start of aggregate formation (C). Experiments shown are representative of n=4. Data are expressed as means ± SD.

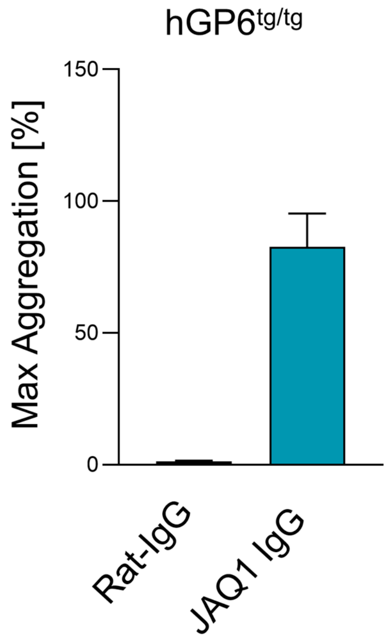

Suppl. Figure S2 . A humanized GP6 mouse line to study the effect of JAQ1 on huGPVI. Analysis of light transmission aggregometry shown in Figure 2F and reported as maximal aggregation. Experiments shown are representative of n=4. Data are expressed as means ± SD.

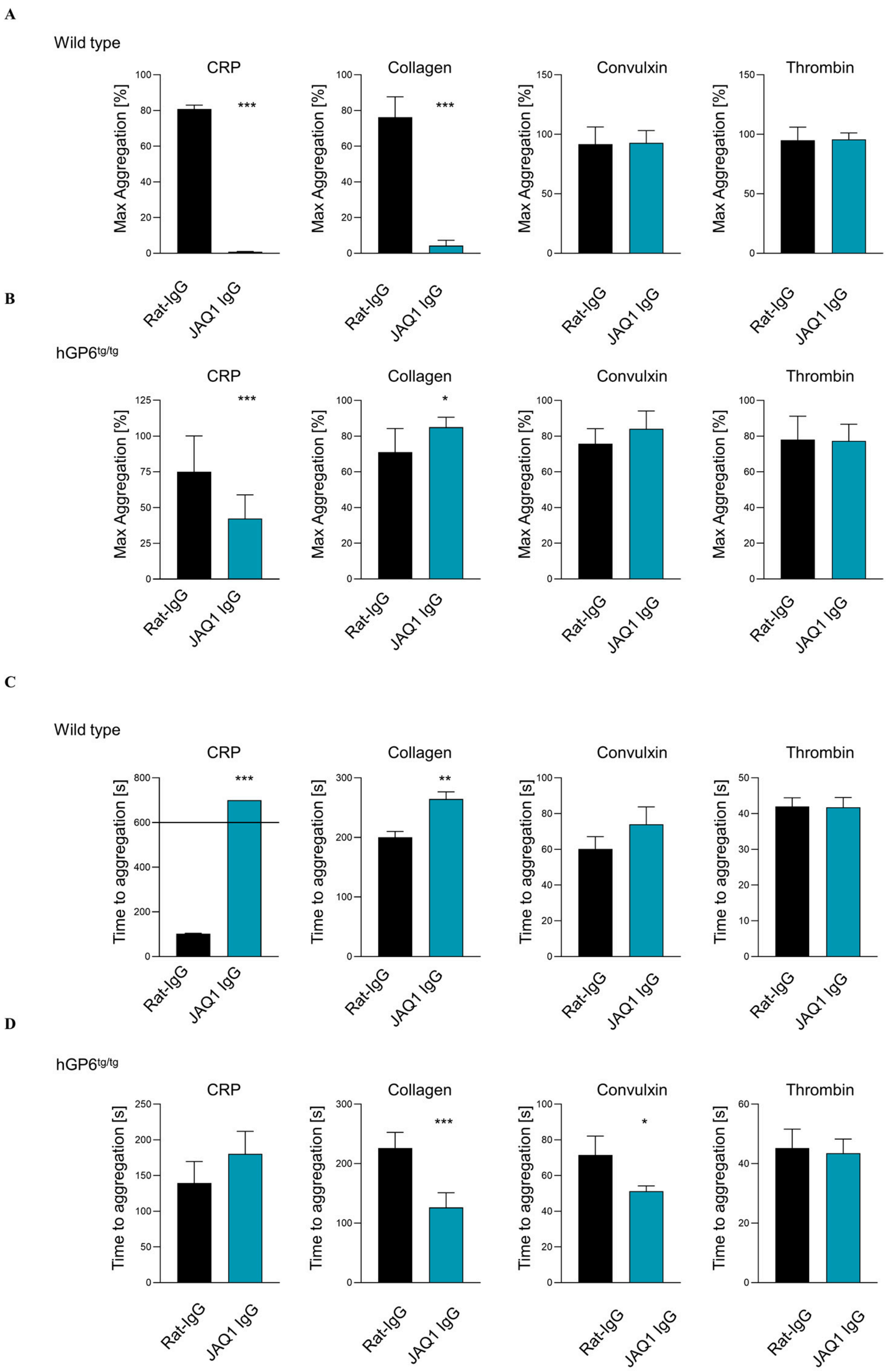

Suppl. Figure S3 . Differential effect of JAQ1 on huGPVI and mGPVI. Analysis of light transmission aggregometry reported as maximal aggregation (A-B) and time to aggregation (time to begin of aggregate formation) (C-D). Experiments shown are representative of at n=4. Data are expressed as means ± SD.

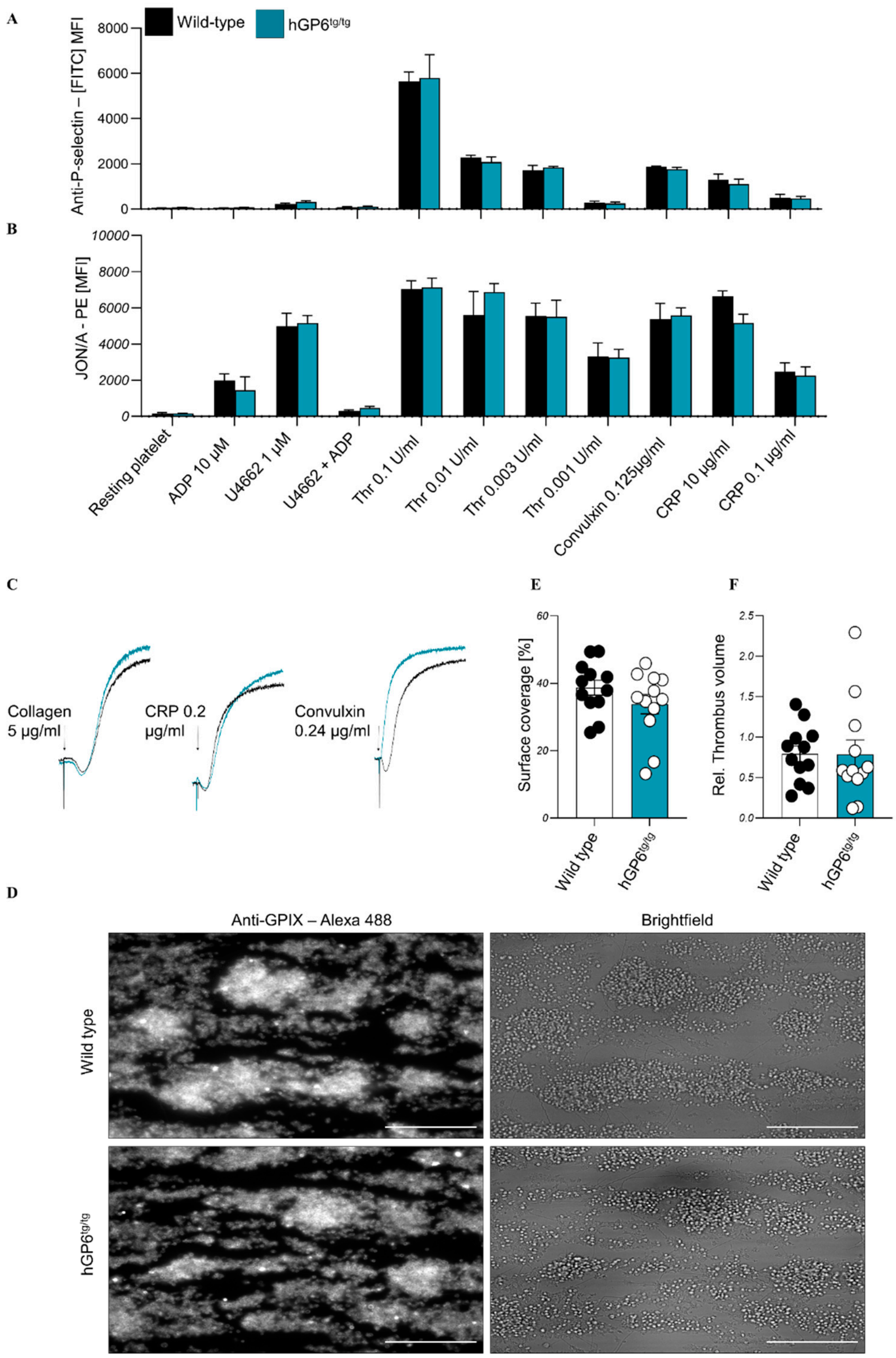

Suppl. Figure S4 . Differential effect of JAQ1 on huGPVI and mGPVI. (A-B) Washed *WT* or *hGP6*$^{tg/tg}$ blood was pre-incubated with the indicated agonists or vehicle. Platelet degranulation (a) and integrin αIIbβ3 activation (b) were measured by flow cytometry. (C) Washed platelets from *WT* or *hGP6*$^{tg/tg}$ were tested in standard aggregomentry using the indicated agonists; aggregation was measured for 10 min. (D-F) Heparinised *WT* or *hGP6*$^{tg/tg}$ blood was tested in a whole blood flow adhesion assay on a collagen-coated surface. Percentage of the covered surface (E) and relative volume of thrombi (F) was analysed based on fluorescence intensity of anti-GPIX^{AF488} derivative. Experiments shown are representative of at least n=4. Flow cytometry data are expressed as means ± SD.

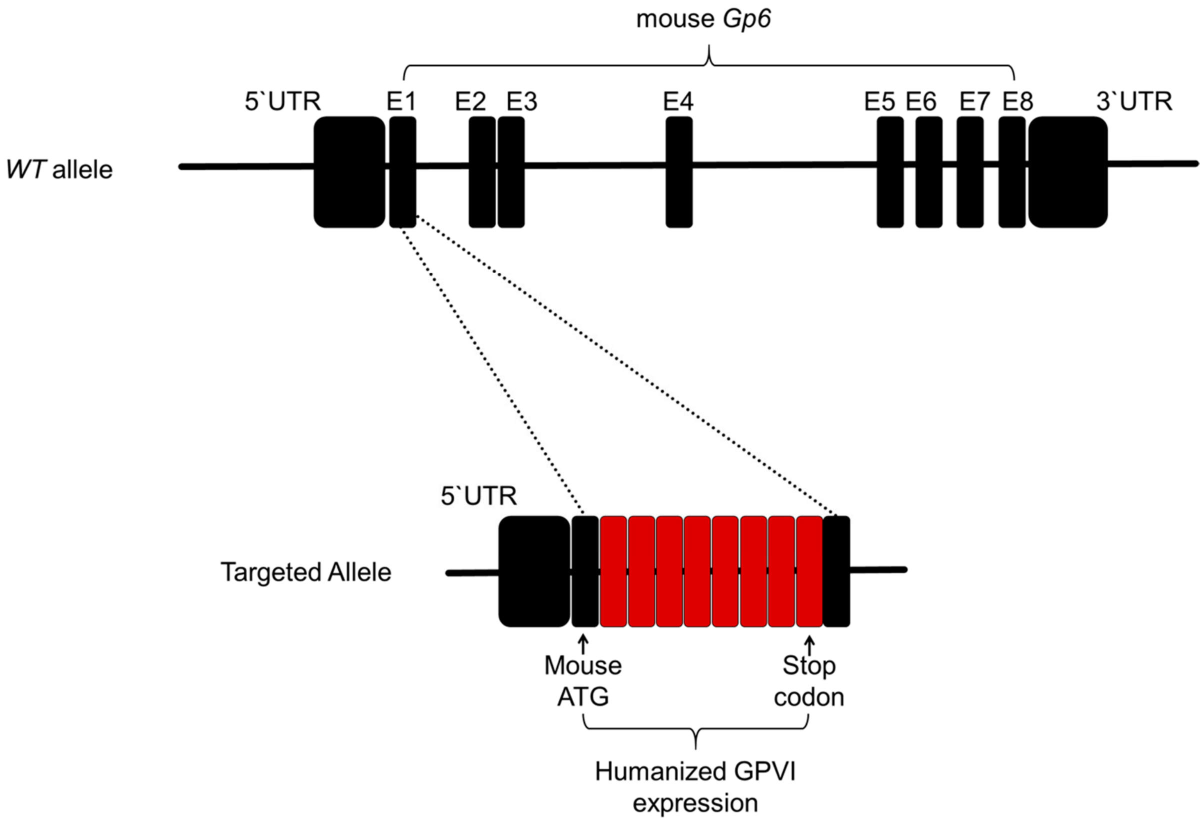

Suppl. Figure S5. For the generation of humanized GPVI (*hGP6*$^{tg/tg}$) mice, the cDNA expressing human GPVI (huGPVI) shown in the figure in red, was inserted at the ATG site of the mouse *Gp6* gene (black) via CRISPR-Cas9 technology. Human *GP6* cDNA carries a stop codon thus allowing only selective expression of huGPVI.
